# Worldwide Impact of Upper Gastrointestinal Disease in Familial Adenomatous Polyposis

**DOI:** 10.3390/diagnostics15101218

**Published:** 2025-05-12

**Authors:** Mahnur Haider, Muaaz Masood, Bryson W. Katona, Carol A. Burke, Gautam N. Mankaney

**Affiliations:** 1 Department of Internal Medicine, Tulane University, New Orleans, LA 70112, USA; 2 Department of Gastroenterology & Hepatology, Virginia Mason Franciscan Health, Seattle, WA 98101, USA; 3 Division of Gastroenterology and Hepatology, University of Pennsylvania, Philadelphia, PA 19104, USA; 4 Department of Gastroenterology, Hepatology, & Nutrition, Digestive Disease Institute, Cleveland Clinic, Cleveland, OH 44195, USA

**Keywords:** familial adenomatous polyposis, gastric polyposis, duodenal polyposis

## Abstract

Familial adenomatous polyposis (FAP) is the most common hereditary colorectal adenomatous polyposis and cancer syndrome which has historically been associated with a near absolute risk of colorectal cancer. However, the morbidity and mortality from colorectal cancer has been greatly diminished by pre-symptomatic genetic testing which identifies affected individuals and by appropriately timed, risk-reducing surgery of the colorectum. Following colorectal surgery, cancer risk beyond the retained rectum or ileal pouch includes other gastrointestinal organs, especially those of the upper gastrointestinal tract. While genotype–phenotype correlations exist for the severity of colonic polyposis, they have not been demonstrated for upper gastrointestinal tract manifestations. We reviewed the impact of ethnicity on the upper gastrointestinal manifestations of FAP by a comparison of published data in patients with FAP from Asian and Western countries. Our main findings demonstrate that following risk-reducing surgery to mitigate colorectal cancer risk, patients with FAP remain at increased risk for upper gastrointestinal polyposis and cancer. The duodenal and gastric phenotype differs between patients with FAP from the West and the East, and all should be followed in a multidisciplinary surveillance program. Following risk-reducing surgery to mitigate colorectal cancer risk, patients with familial adenomatous polyposis remain at increased risk for upper gastrointestinal polyposis and cancer. The duodenal and gastric phenotype differs between patients with FAP from the West and the East, and all should be followed in a multidisciplinary surveillance program.

## 1. Introduction

Familial adenomatous polyposis (FAP) is the most common hereditary colorectal adenomatous polyposis and cancer syndrome which has historically been associated with a near absolute risk of colorectal cancer. However, the morbidity and mortality from colorectal cancer has been greatly diminished by pre-symptomatic genetic testing which identifies affected individuals and by appropriately timed, risk-reducing surgery of the colorectum. Following colorectal surgery, cancer risk beyond the retained rectum or ileal pouch includes other gastrointestinal organs, especially those of the upper gastrointestinal tract. While genotype–phenotype correlations exist for the severity of colonic polyposis, they have not been demonstrated for upper gastrointestinal tract manifestations. The greatest morbidity and mortality in patients with FAP is from upper gastrointestinal cancer, in particular, duodenal and ampullary cancer. The stage of duodenal polyposis correlates with cancer risk and increases with age. Data suggest patients with FAP from Asian countries have lower rates of duodenal polyposis, but not ampullary cancer, compared to patients with FAP from the West. The elevated risk for FAP-related gastric cancer (FAP GC) in the East has been well documented. FAP GC was not recognized as an extracolonic cancer risk in the West until 2017. The features of FAP GC differ between patients from the East and the West. As patients with FAP live longer following colorectal surgery, their risk for upper gastrointestinal tract polyposis and cancer rises, but the risk and features of disease differ by ethnicity, which may subsequently impact approaches to upper gastrointestinal tract surveillance. We reviewed the impact of ethnicity on the upper gastrointestinal manifestations of FAP by a comparison of published data in patients with FAP from Asian and Western countries (Table 1). We demonstrated that patients with FAP remain at increased risk of upper gastrointestinal polyposis regardless of ethnicity though their phenotypes vary.

## 2. Methods

A comprehensive literature search on PubMed using a combination of keywords related to familial adenomatous polyposis (FAP), Barrett’s esophagus, gastric polyps, fundic gland polyps, gastric cancer, duodenal polyposis, jejunal and ileal polyps, and duodenal cancer was conducted. Reference lists of the selected articles were also screened for additional studies. Articles that were unrelated to the topic were excluded. Articles were removed if they were not available in English or full text. Duplicate articles were excluded. Systematic reviews, meta-analyses, and randomized controlled trials were assigned a high priority. Eastern and Western studies were determined by the country of origin in order to explore potential regional differences in findings and clinical practice. A total of 85 articles were included in the final review.

### 2.1. Esophagus

The esophagus is typically not considered a “classic” location for familial adenomatous polyposis (FAP)-associated pathology. The lack of involvement of the esophagus is likely related to the distinct molecular differences between the squamous epithelium-lined esophagus compared to the columnar epithelium found throughout the rest of the gastrointestinal (GI) tract, with esophageal squamous cell carcinomas showing significantly lower mutation rates in *APC* compared to both esophageal and gastric adenocarcinoma on somatic next generation sequencing in the general population [27]. The only esophageal lesion reported with any frequency in FAP is Barrett’s esophagus (BE), which involves replacement of the squamous epithelium of the esophagus with the columnar-lined intestinal type mucosa harboring intestinal metaplasia [28]. BE is associated with a significantly increased risk of esophageal adenocarcinoma, a transition that involves the *APC*-regulated Wnt signaling pathway [29].

There have been several small reports from the United States (U.S.) and Europe that suggest an association of BE with FAP [1,30,31,32]. However, the magnitude of this association remains uncertain. The most recent and largest study to date showed that 16% of patients with FAP had BE, a significantly increased incidence compared to age-matched controls [1]. Additionally, the average age of BE diagnosis in FAP was 37.8 years, which is strikingly lower than the average age of 57.5 years for BE diagnosis in the sporadic BE control group [1]. Given the limited published data, the overall incidence of BE in FAP currently remains uncertain, and larger, multi-center cohort studies are needed. There are no studies from Asia that explore an association between BE and FAP. Future studies should explore a genotype–phenotype correlation between the location of the disease-causing *APC* alternation and BE.

If BE is identified in an individual with FAP, the BE should be treated as per the standard of care guidelines which may include both a proton pump inhibitor (PPI) and endoscopic surveillance. Most patients with BE are treated with PPI therapy given the documented reductions in risk of esophageal adenocarcinoma in BE associated with PPI use [33]. In FAP, the main consideration is that PPIs may increase fundic gland polyp growth [34]. As fundic gland polyposis is common and often advanced in FAP (refer to Section 2.2 Stomach), additional fundic gland polyp growth may potentially interfere with effective GC surveillance. As for BE surveillance, patients with FAP should routinely undergo upper GI surveillance at least every 3 years with shorter intervals utilized for gastric or duodenal neoplasia which requires follow-up. This interval will be sufficient for monitoring non-dysplastic BE. However, the management of dysplastic BE may require more frequent upper endoscopy than what would be required for FAP alone. Finally, if surgical therapy for BE is to be pursued in an individual with FAP, the remaining burden of upper GI neoplasia, as well as the need for continued upper GI surveillance, has to be taken into account when determining what type of surgical procedure is best for the patient.

### 2.2. Stomach

Proximal gastric polyposis is near universal in patients with FAP, and the fundic gland polyp is the most common gastric lesion in all populations [2,3,7]. In a review of 75 consecutive patients from the West at a mean age of 44.2 years who had endoscopic biopsies, 88% of subjects had polyps. All polyps were histologically consistent with FGP, of which 38% had low-grade dysplasia and 3% had high-grade dysplasia [7]. A Korean and Japanese study evaluating the prevalence of upper gastrointestinal pathology found that 38 out of a total 59 (64.4%) and 63 out of a total 122 (55.3%) patients with gastric polyps had FGPs, respectively [2,3]. The mean age of FGP diagnosis was 33.6 years in the Japanese study while the mean age of all participants was 34 years in the Korean study.

Other gastric neoplasms found in the stomach of patients with FAP include gastric adenomas, pyloric gland adenomas, and hyperplastic polyps. In the West, the prevalence rates of gastric adenomas and pyloric gland adenomas range from 9% to 23% and 4.3–6%, respectively [8,9,10]. Gastric adenomas are found in 33 of 225 (14.7%) Japanese and 21 of 59 (35.6%) of Korean patients with FAP [2,3].

### 2.3. Gastric Cancer

The cumulative incidence rates of FAP GC have historically differed between the East and West, ranging from 2.6–13.6% [2,4,5,6] in Japan and 4.2% in Korea [3] to only 0.6% in the US [11]. In fact, GC was not considered a manifestation of FAP in the West despite being the leading cause of extra-colonic cancer in Japan and Korea until recently [35]. The higher background population incidence rates of GC in Japan and Korea compared to the West have been attributed as the cause of the increased FAP GC rates in Japanese and Korean cohorts. Patients with FAP from Japan and Korea are 7 and 2.4–4.7 times more likely to develop GC than the general population, respectively [4]. Data from recent publications on FAP GC in Western cohorts now recognize it as an extra-colonic manifestation of FAP, with the standardized incidence ratio of 760 in a large US registry compared to the general population. Of note, 1.3% of the cohort had GC [36].

In a study of eighty patients with FAP, 24% had sessile gastric polyps. Patients with sessile gastric polyps were noted to be older, more likely to have a family history of GC, have white mucosal patches in the proximal stomach and antral polyps compared to patients without a gastric neoplasm [37]. In fact, FAP GC has been shown to be the leading all-cause mortality in patients with FAP [38]. Data in FAP patients from Brazil report that 3.9% develop FAP GC [8]. The reason for this rise in incidence is unclear but it is concerning as the genotype has not changed.

The average age of GC does not differ between the East and the West, ranging from 50.3–58 years in the West and 48–58 years in the East. Both sexes are equally affected with adenocarcinoma on pathology [2,3,4,5,8,36]. The stage and endoscopic presentation of GC, however, differs between the two regions. In Japan, most cancers are multicentric as 60% of patients with FAP versus 2–19% in the general population are distributed throughout the stomach, well-differentiated, and present at an early stage [5]. In the United States, GC is unifocal and located proximally [36,39]. Before it was recognized as a cancer risk in FAP, GC was diagnosed at an advanced stage and had a poor prognosis. Due to the increasing recognition of risk of FAP GC and recommendations for early detection and management, FAP GC is now being diagnosed at a much earlier stage in the US [36]. In a study of 337 patients with FAP who underwent 2052 endoscopies from 1989 to 2023, the 10-year cumulative incidence of FAP GC was 0% with no polyps, 1% with polyps, 6% with low-grade dysplasia, 11% with polyps ≥2 cm, and 20% with high-grade dysplasia [40]. Novel work identifying the endoscopic and histologic features in the stomach of patients with FAP who develop GC and recommendations for intensified endoscopic surveillance and endoscopic intervention have led to this change [36,39].

Risk factors for FAP GC differ between nations. *H. pylori* (HP), a known gastric carcinogen, is associated with GC in Japanese patients with FAP, where population rates are also elevated, but a low prevalence rate of HP in the West is observed and is not linked to FAP GC [41]. There have been increased reports of *H. pylori*-naïve gastric neoplasms in Japan [42].

For some manifestations of FAP, correlations can be made between the phenotype of the disease and the location of the pathogenic variant on the *APC* gene. A common example is that pathogenic variants on the distal ends of the gene (5′ or 3′) are associated with attenuated colonic polyposis, or <100 cumulative colonic adenomas, while pathogenic variants in between are associated with classic polyposis, or ≥100 cumulative colonic adenomas. In Japan, classic FAP is associated with an increased prevalence of fundic gland polyps as well as gastric adenomas compared to attenuated FAP. Furthermore, development of gastric adenomas is linked to pathogenic variants between codons 1450–1564, which are located in the center of the gene, and somatic mutations between codons 1554–1556 have been demonstrated in half of FAP-associated fundic gland polyps and gastric adenomas [43]. In contrast, pathogenic variants in codons 564–1465 are associated with gastric adenomas, but not fundic gland polyps [17,44]. However, in a study of 234 patients with FAP in Japan, the endoscopic phenotype was associated with the histopathologic phenotype, but not to the germline variant [45].

The precursor lesion for GC remains to be confirmed although it is likely a gastric adenomatous neoplasm in both Eastern and Western patients with FAP [2,36,39]. Experts suggest FAP GC arises from gastric adenomas or pyloric gland adenomas as they have been found prior to or at the time of the FAP GC diagnosis. In a retrospective review of 161 gastric polyps from ten patients with GC from a large US registry, all cancer cases arose from gastric adenomas, pyloric gland adenomas, or polyps with mixed features of the two (unpublished data). The endoscopic features, which have been discovered in higher prevalence in patients with FAP GC, include a carpeting of polyps [46], defined as no intervening normal mucosa in an area of proximal polyposis, proximal mounds of polyps, and large >1–2 cm gastric polyps [39]. Carpeting of polyps is mostly comprised of fundic gland polyposis amidst which other gastric neoplasms may not be easily observed. A white mucosal patch (WMP), which is an area of pale colored mucosa in the proximal stomach, has been visualized in the stomach of Western patients with FAP and is associated with the presence of high-risk pathology and GC anywhere within or outside of the WMP in the proximal stomach [47]. In a Japanese series, no patient had fundic gland polyps preceding their GC while a Japanese case report described antral adenomas found over years of endoscopic surveillance in a patient who later developed multifocal antral adenocarcinoma [5,48]. A review of 303 Japanese patients with FAP found that GC was significantly more common in patients with a gastric adenoma than those without. The prevalence rate of gastric adenomas was higher in those with GC (36.4%) compared to those without GC (12.4%) [2]. WMP, gastric mounds, and polyp carpeting associated with GC have not been described in Japan and Korean patients with FAP.

We recommend the frequency of upper endoscopic surveillance to be determined by the duodenal and gastric phenotype, whichever of the two is more advanced, in both the East and West. Individuals with gastric mounds, WMP, carpeting, large ≥1 cm polyps, pyloric gland adenomas, gastric adenomas, and hyperplastic polyps should have an annual upper endoscopy. If dysplasia is present, excluding fundic gland polyps with low-grade dysplasia, the intervals should be every 6 months with consideration for gastrectomy. In Japan, antral adenomas are small and depressed compared to fundic gland polyps, which protrude. In the West, the majority of, if not all, polyps occur proximally in the fundus or body, which can make the stomach difficult to examine. We have developed criteria for endoscopically diagnosis concerning pathology or what we suspect are precursor lesions with high-definition white light (HDWL) and narrow-band imaging (NBI). The concerning lesions have open circular pits, appear similar under NBI and HDWL, and have a nodular surface [49]. The gastric surface should be carefully examined, particularly in areas of large polyps or carpeting. We diagnosed one GC located under a mound of polyps with endoscopic ultrasound. For stomachs with one or more large mounds, or dense areas of polyposis, we recommend endoscopic ultrasound as surface examination and biopsies may be superficial and unrevealing. The European FAP consortium has proposed personalized guidelines for endoscopic surveillance and treatment for patients with FAP [50].

## 3. Non-Ampullary Duodenum

### 3.1. Duodenal Polyps

The duodenum is the second most common site of adenomatous polyposis in FAP and is a leading cause of cancer once colectomy has been performed. In Western populations, duodenal polyposis develops in nearly 100% of patients with FAP. Duodenal adenomas have been noted to occur approximately 15 years after colonic adenomas [51]. Data show an age-associated increase in the cumulative incidence of duodenal polyposis, from approximately 40–65% at the age of 40 years to 90–98% by the 8th decade. Other data in these populations suggest a much higher prevalence of duodenal polyposis at younger ages [18,19]. Large FAP registry studies reported duodenal polyposis in 86–90% with FAP at a mean age of 40 to 44 years [7,20,52]. Other data in Western populations show duodenal adenomas are detected as early as in adolescence. In the two largest cohorts of pediatric patients with FAP in the US, duodenal adenomas were reported in 33–52% at an average of 13.5–15 years of age [17,21]. Similarly, a multi-center study from France found duodenal adenomas in 23 of 54 (43%) children with FAP undergoing EGD, with the youngest being 6.9 years old [22].

In Eastern cohorts with FAP, similar variability in the reported occurrence and age at diagnosis of duodenal polyposis is noted. In two single center studies from Japan, duodenal polyps were reported in 47 of 77 (61%) patients at a mean age of 39 years, and in 104 of 130 (80%) patients who were 34 years old when FAP was diagnosed. Of note, the age at diagnosis of duodenal polyposis was not reported [12,13]. A multi-center study of 303 patients from Japan detected duodenal polyps in 39% at a mean age of 50 years [2]. The prevalence of duodenal polyps was 8.8% at a mean age 34 years in a study in 148 South Korean patients with FAP undergoing a baseline EGD [3]. Of the eighty-seven patients (59%) who underwent a surveillance EGD at a mean follow-up of 5 years, 10 patients (11.5%) had newly-developed duodenal polyps. A more recent study from South Korea in 215 patients with FAP, followed between 1991 and 2019, described duodenal adenomas in 86 of 208 (41%) patients who had an EGD [14]. The mean age of FAP diagnosis was 29 years (IQR 23–37 years) with a mean of 78 months of follow-up (IQR 26–121 months). In a study from Iran, 7 of 28 patients (25%) at a mean age of 37.4 years were diagnosed with duodenal polyps [15]. Registry data in 108 Chinese patients observed duodenal polyposis in 4 (3.7%) patients at a median diagnosis of 43 years [16]. Summation of these data suggests that the prevalence of duodenal polyposis is less in Asian versus Western patients with FAP.

### 3.2. Staging of Duodenal Polyps

The Spigelman staging (SS) system is a 5-stage classification system (0–IV) of duodenal polyposis severity [52]. Points are assigned to different ranges of duodenal polyp size, number, histology, and degree of dysplasia and added to calculate a score. The highest SS IV, is associated with the greatest risk of duodenal cancer [18,19,25]. The majority of studies conducted in the West have demonstrated a progressive nature of duodenal polyposis and an increase in SS with age. In a prospective, Finish cohort of 52 of 71 patients with FAP, who had more than one EGD over 11 years of follow-up, progression of SS was noted in 74% of patients and the cumulative lifetime risk for SS IV polyposis was 30% [53]. Few patients underwent endoscopic therapy of duodenal polyps in the study. In a multi-center cohort of 261 patients from Nordic countries followed over a median of 14 years, the cumulative lifetime risk of SS IV polyposis was 35% [26]. SS improved in 12%, was unchanged in 34%, and worsened in 44% of patients in the study. The authors suggest the apparent improvement in SS may result from duodenal adenoma resection, but no details were provided in the study. In a study of 49 patients with FAP, prophylactic duodenal polypectomies were noted to be relatively safe compared to papillectomies which were associated with significant adverse events [54]. In a large US natural history study of 114 patients with duodenal polyposis with two or more surveillance EGDs followed over a mean of 51 months, 26% of duodenal polyps progressed in size, 32% in number, and only 11% in histology [23]. In a study of 58 patients with FAP who had multiple duodenal adenomas, intensive downstaging polypectomy cold snare or forceps polypectomy and underwater endoscopic mucosal resection revealed that the Spigelman stage was significantly decreased at the 1-year follow-up endoscopy (*p* < 0.001), with downstaging noted in 39 patients (71%) [55].

Duodenal polyposis did not change in 85% of 41 Japanese patients with FAP, which included 98% with SS II or III polyposis at baseline, over a median of 10.6 years [22]. No mention was made if endoscopic therapy was applied in those patients. Similarly, no progression in SS was noted in 73% of Korean patients between the baseline and a two-year follow-up EGD, though 55% underwent endoscopic treatment of adenomas [14]. In the untreated group, SS significantly increased whereas in the treated group, SS significantly decreased. Upstaging after two years was significantly higher in the untreated group (27%) than in the treatment group (0%). Studies in Asian populations depict a slower progression of duodenal polyposis than that reported in Western studies. Factors that are likely to contribute to variation in the cohorts studied include age, duration of follow-up, size of cohort, approach to surveillance, use of endoscopic therapy, and referral bias.

### 3.3. Duodenal Cancer

Duodenal cancer is a dreaded complication of FAP with a high risk of mortality. In a ten-year study of 114 patients followed in an EGD surveillance program in a registry in England, 5% developed duodenal adenocarcinoma at a median age 67 years [25]. Similarly, 7% of Nordic patients in a surveillance program developed duodenal cancer at a median age of 56 years [26]. Both studies confirmed the risk of duodenal cancer is highest in patients who have SS IV duodenal polyposis at baseline. While SS IV is predictive of duodenal cancer risk, a study from a US FAP Registry demonstrated SS IV polyposis was absent in half of patients with FAP who had duodenal cancer [23]. The study found that only large duodenal polyp size and HGD, but not the number of duodenal polyps, were positively associated with duodenal cancer.

The occurrence of duodenal cancer in 114 patients in the US subjected to two or more systematic surveillance EGDs over 51 months was 0.8% [23]. While systematic surveillance was applied to patients in all studies [23,25], endoscopic therapy of duodenal polyps was not applied routinely. The occurrence of duodenal cancer in two Asian cohorts with FAP was 4.2% in Korean patients [3] and 1% in Japanese patients [12] over an observation period of five and ten years, respectively. In another study from Japan, duodenal cancer occurred in 7.7% of patients at a median age of 50 [2].

In Japan, the association between colonic and duodenal phenotypes was examined and a higher duodenal adenoma risk of 42.5% was noted in patients with classical FAP compared to attenuated FAP, which was associated with a 23.5% duodenal adenoma risk [2]. A similar correlation was seen in a cohort of patients from a registry in Utah, whereby modified SS duodenal polyposis of II or greater was found in 88% of patients with classic FAP and in 48% of patients with attenuated FAP [56]. However, no conclusive *APC* genotype–phenotype correlation has been confirmed in Western or Eastern cohorts [2,14,19,57].

### 3.4. Duodenal Polyp Surveillance

One Japanese and many Western guidelines on the use of EGD surveillance in patients with FAP have been published [58,59,60,61,62]. While the Japanese statement states no consensus has been reached on this topic, they suggest the SS can be referred to for guidance on duodenal polyp surveillance [58]. Most Western statements recommend starting surveillance with endoscopy around ages 20–25 [59,60,62] or 25–30 years [61] and repeat endoscopy at an interval according to the SS, with consideration of prophylactic duodenectomy for individuals with SS IV polyposis in lieu of expert surveillance every 3–6 months. Importantly, visualization of the papilla, in addition to the duodenum, is an important component of high-quality EGD surveillance in patients with FAP.

## 4. Ampullary and Periampullary Duodenum

The risk of ampullary adenocarcinoma can be more than 100-fold greater in Eastern and Western patients with FAP than in the general population [12,14,23,63]. Lesions of the ampulla are most often adenomas and adenocarcinomas, while lipomas, leiomyomas, hemangiomas, and leiomyofibromas are infrequently observed. Ampullary adenomas in FAP, usually discovered on routine surveillance EGD, are usually asymptomatic (unlike their sporadic counterparts), develop 10–20 years after colonic polyposis onset, and rarely are the presenting feature of FAP [51]. Studies from Japan indicate that ampullary adenomas are detected on endoscopy between 31.7 and 40 years of age. Though details on ampulla morphology are not provided, we assume that the authors are describing endoscopically visible adenomas [12,14,64]. In a review of Western literature, the age at ampullary adenoma detection is not provided. However, if the ampulla appeared adenomatous, histologic confirmation of adenoma was demonstrated in 89% of patients [23,63]. Furthermore, 36–54% of FAP patients have biopsy-proven adenomatous histology even in the presence of a normal appearing ampulla, and not all centers obtain biopsies of the ampulla for endoscopically normal tissue [23,24]. Eastern centers demonstrated similar prevalence rates with 25% and 36% of patients with FAP from Korea and Japan having ampullary adenomas, respectively [12,14].

Ampullary adenomas are managed less aggressively in Western and Eastern patients with FAP compared to if they were identified sporadically, as they slowly progress in size or histology [23,64,65,66]. A Japanese study demonstrated that out of twelve patients with ampullary adenomas, only two progressed in size over 8.5 years of follow-up [14]. Similarly, 95/110 (86%) of patients had no statistically significant progression in morphology of the papilla over a mean 50.4 months of follow-up in a US study [23]. Out of seven ampullary cancer cases in a United Kingdom registry, the median Spigelman stage at the time of first endoscopy and cancer diagnosis remained unchanged at 2 years [23,67]. In a study of 143 patients with FAP and ampullary adenomas with a median follow-up of 7.8 years, most patients did not have clinically significant progression and ampullary cancer was rare. Factors which were associated with clinically significant progression included male gender, abnormal papilla at AA detection, cholecystectomy, and a history of extracolonic malignancy [68].

Advanced histology, such as villousity and HGD, are observed more frequently in individuals who develop duodenal adenocarcinoma [56]. Management options for patients with ampullary adenomas include observation, endoscopic ampullectomy, or surgery and are based on size, histology, symptoms, patient characteristics, as well as progression. While chemoprevention has been studied extensively for its role in reducing duodenal adenoma burden, studies have not specifically focused on ampullary neoplasia endpoints, therefore, chemoprevention should not be considered as a definitive treatment strategy for ampullary neoplasia in FAP [69]. There are no guidelines specifically delineating when to utilize definitive therapy for the ampulla. However, it should be considered for an adenomatous ampulla that leads to symptoms, is greater than 10 mm in size, has evidence of HGD, or is growing at an increased rate [62]. The degree of duodenal polyposis may also influence the feasibility of endoscopic therapy, as surgery may be the preferable option if advanced duodenal polyposis is also present.

Guidelines currently recommend visualization of the ampulla during EGD surveillance [59,60,62,70,71] with either a side-viewing endoscope or a forward-viewing endoscope with a clear cap, which provides adequate visualization of the ampulla in up to 95% of patients [72,73]. Current FAP surveillance guidelines do not account for the histology of the ampulla, and the ASGE recommends against routine biopsy of a normal appearing ampulla given concerns for pancreatitis [59]. In a large series of 273 patients with FAP, only two (0.73%) developed post-procedure pancreatitis after biopsy of the ampulla, one of whom had a history of chronic pancreatitis in the setting of alcohol use disorder [20]. Inclusion of the biopsy data led to a more advanced SS in 13.2% of patients, and one patient with a normal appearing ampulla had villous histology with high-grade dysplasia, which highlights the value of obtaining biopsies. We recommend visualization of the ampulla with biopsy.

### 4.1. Endoscopic Ampullectomy

Endoscopic ampullectomy is becoming increasingly utilized for the treatment of ampullary adenomas. One advantage of endoscopic ampullectomy is that it obviates the need for invasive abdominal surgery, an important consideration in FAP given concern for post-surgical desmoid formation [74]. It is associated with less morbidity and mortality compared to surgery [75] and has >90% success rates [76]. Up to 58% of patients with FAP have recurrent or residual [77] ampullary adenomas, though other studies have shown lower estimates for recurrence [66,78,79]. A recent cumulative analysis of 28 reports of endoscopic ampullectomy totaling 1569 patients demonstrated a cumulative success rate of 80% with a 12.1% recurrence rate [76]. In a multicenter study with 100 patients with FAP and 157 patients with sporadic ampullary adenomas, recurrence of ampullary adenomas after endoscopic papillectomy occurred in 48% of patients with FAP and 36.9% of patients with sporadic ampullary adenoma. Ampullary adenoma size, periampullary extension, and biliary duct dilation increased the risk of recurrence whereas en bloc resection decreased the risk of recurrence [80]. While guidelines by the National Comprehensive Cancer Network support endoscopic ampullectomy, the topic is not addressed in Japanese guidelines while guidelines from the United Kingdom recommend against it due to risks [58,60,62].

The goal of endoscopic ampullectomy should be en bloc resection of the ampulla. Endoscopic ampullectomy is associated with a post-procedure adverse event rate of 10.2% [66]. A cumulative analysis of multiple endoscopic ampullectomy studies demonstrated complication rates of bleeding, pancreatitis, perforation, stenosis, and cholangitis being 10.2%, 12.3%, 3.3%, 2.9%, and 4.3%, respectively [76]. Empiric placement of a pancreatic duct stent can be considered to minimize post-procedure pancreatitis risk [81,82], but not all studies demonstrated a benefit [76]. After endoscopic ampullectomy, patients should remain in an endoscopic surveillance program given the recurrence rates.

### 4.2. Surgical Treatment of Ampullary Lesions

Surgery can be considered for endoscopically unresectable ampullary lesions, ampullary adenocarcinoma, if there is substantial ingrowth of the adenoma into the bile/pancreatic ducts, if there is advanced stage IV duodenal polyposis, or in select circumstances after recurrence following endoscopic resection. Surgical options include ampullectomy via a transduodenal approach as well as pancreaticoduodenectomy. Transduodenal surgical ampullectomy has been previously utilized. However, given the improvement in endoscopic ampullectomy techniques, this procedure is infrequently performed [83]. Pancreaticoduodenectomy provides definitive therapy for an ampullary adenoma, eliminating the risk for local recurrence as well as the need for future surveillance of the ampulla. However, this procedure is associated with increased risks [84].

### 4.3. Chemoprevention of Duodenal Polyps

In the past, sulindac and erlotinib combination therapy was noted to decrease duodenal polyp burden, but the rate of adverse events was relatively high. In a randomized controlled trial of 46 patients with FAP, erlotinib 350 mg once weekly for 6 months significantly decreased the duodenal polyp burden and was associated with generally lower grade adverse events. Further studies are warranted to explore agents to prevent cancer for patients with FAP.

## 5. Jejunum and Ileum

The clinical relevance of jejunal and ileal polyps is not known. In a study of 102 patients with FAP, the prevalence of jejunal adenomas was 11.8%. Eleven out of twelve patients with jejunal adenomas also had duodenal adenomas [8]. Wood et al. reported in a study of 66 patients with FAP, tubular adenomas were present in the small bowel in 89% of patients and one patient was noted to have invasive carcinoma of the small bowel [9]. Jejunal and ileal polyps have been associated with the development of duodenal polyps. In a study of 15 patients with FAP who underwent capsule endoscopy for routine upper endoscopic surveillance, 60% had small intestinal polyps. The study concluded that CE should be performed in FAP patients with Stage III and IV duodenal disease [85]. Additionally, jejunal and ileal polyps have also been associated with patients with FAP who have had a duodenectomy. Bhatt et al. revealed that 59% of patients with FAP after duodenectomy had jejunal polyposis. While jejunal polyposis was advanced in 21% of patients, it rarely required surgery [86]. In a study of 119 patients who underwent duodenectomy, 41% were diagnosed with jejunal adenomas after duodenectomy with a higher proportion in patients who underwent pancreas-preserving total duodenectomy compared to pancreatoduodenectomy. Two patients developed jejunal cancer (2%) and all but one patient with postoperative gastric/jejunal cancer died [87].

Several studies have explored diagnostic modalities for jejunal and ileal polyps in patients with FAP. Caspari et al. compared CE and MRI for small intestinal polyps in patients with FAP, polyps >15 mm were detected similarly whereas smaller polyps were detected much more often with CE. The location of the detected polyps and size of the polyps was more accurate with MRI compared to CE [88]. Schulmann et al. demonstrated that CE may be of clinical value in selected patients with FAP [89]. Additional studies have confirmed the role of VCE for small bowel polyps in patients with FAP [90,91,92,93,94,95,96]. When compared to MR enterography, CE detected small-sized polyps in the small intestine whereas MR enterography provided additional extraintestinal information, i.e., formation of desmoid tumor on the anterior abdominal wall [97].

Alderlieste et al. demonstrated that clinically significant jejunal polyposis in FAP was rare, even in advanced duodenal disease, and stated that routine jejunoscopy does not seem warranted [98]. Pertaining to the role of double-balloon enteroscopy, Sekiya et al. documented that endoscopic resection of duodenal and jejunal polyposis using DBE in patients with FAP was safe and effective [99]. The diagnostic yield of DBE was reported to be similar to that of intraoperative enteroscopy for small intestinal polyps in patients with FAP [100].

Current guidelines do not recommend routine small bowel imaging in all patients with FAP. Polyps in the jejunum and ileum have been more commonly reported compared to jejunal or ileal cancer. However, small bowel screening may be considered in patients with advanced duodenal polyposis and those who have had a duodenectomy. Video capsule endoscopy or MR enterography are the preferred modalities for screening. If there is a positive finding on VCE or MR enterography, a deep enteroscopy can be considered for therapy. Additionally, a deep enteroscopy may be needed if patients present with symptoms concerning a small bowel pathology and before duodenal surgery.

## 6. Conclusions

We recommend the initiation of EGD surveillance at 20–25 years of age with close examination of the stomach and the duodenum. Surveillance intervals should be based on the most severely affected organ, the stomach or duodenum. If there are features suggestive of advanced histology in the stomach or duodenum, management options should be approached in a multidisciplinary manner with a surgeon and gastroenterologist. We recommend that all duodenal polyps ≥5 mm are resected with cold snare polypectomy, when possible, to minimize fibrosis. We also recommend limited use of tattoo outside of a clinical trial to prevent fibrosis. Patients with stage IV polyposis or polyps that cannot be resected on endoscopy should be referred to a surgeon for either a pancreaticoduodenectomy or pancreas-sparing duodenectomy. Following surgery, the proximal small bowel remains at risk for polyposis and endoscopic surveillance should be continued [86]. Finally, chemoprophylaxis may be used to manage duodenal polyposis in a select group of patients as no drugs to date have been shown to delay surgery or decrease mortality from FAP (Table 2) [69]. Current gaps in the literature include validated scales to assess upper gastrointestinal polyposis, an understanding of the precursor lesion to gastric cancer and its progression, and development of tools for cancer mitigation beyond endoscopy and surgery. Future studies should validate endoscopic criteria for optical diagnosis of gastric neoplasia, as well as the Spigelman stage, and explore reasons for the rise in GC in patients with FAP from the West, elucidate the dysplasia–carcinoma pathway as well as research differences in phenotype between East and West. Finally, more studies are needed to identify chemoprophylactic agents to prevent duodenal and gastric cancer, for which there currently are none. In summary, both Western and Eastern patients with FAP are at risk for gastric and duodenal cancer and should be followed in a multidisciplinary surveillance program.

## Figures and Tables

**Table 1:** Relationship between ethnicity and upper gastrointestinal manifestations in familial adenomatous polyposis.

Relationship Between Ethnicity and Upper Gastrointestinal Manifestations in Familial Adenomatous Polyposis			
Organ	Rates in the Eastern Populations	Rates in the Western Populations	Surveillance Strategies and Management Approach
Esophagus	No studies from Asia	Barrett’s esophagus Incidence: up to 16% [1]	EGD every 3 years, treat with proton pump inhibitors Shorter intervals for Barrett’s esophagus with dysplasia
Stomach	Fundic gland polyposis Prevalence: Korea 64.4% Japan 55.3%) [2,3] Gastric adenomas Prevalence: Japan 14.7%, Korean 35.6% [2,3] Gastric cancer Incidence: Japan 2.6–13.6% Korea 4.2% [2,3,4,5,6]	Fundic gland polyposis Prevalence: (88%) [7] Gastric adenomas Prevalence: (9–23%) [8,9,10] Pyloric gland adenomas Prevalence: (4.3–6%) [8,9,10] Gastric cancer Incidence: 0.6% [11]	Determined by duodenal and gastric phenotype, whichever is more advanced If high-risk gastric features are present *: annual endoscopy If dysplasia is present, shorter intervals based on type and degree If >1 large mounds or dense area of polyposis: consider EUS Refer to the European FAP consortium guidelines
Non-ampullary duodenum	Duodenal polyposis Variability in the incidence and prevalence [2,3,12,13,14,15,16]	Duodenal polyposis Variability in the incidence and prevalence [7,17,18,19,20,21,22]	EGD at age 20–30, repeat endoscopy according to Spigelman stage ◦ Duodenal polyps ≥5 mm should be resected with cold snare polypectomy when possible to minimize fibrosis ◦ Consider prophylactic duodenectomy in Spigelman stage IV in lieu of endoscopic surveillance every 3–6 months
Ampullary and peri-ampullary duodenum	Ampullary adenomas Prevalence: Korea 25%, Japan 36% [12,14] Duodenal cancer Occurrence: Korea 4.2%, Japan 1–7.7% [2,3]	Ampullary adenomas Prevalence: 36–54% [23,24] Duodenal cancer Occurrence: 0.8–5% [23,25,26]	EGD with biopsy of ampulla as indicated at age 20–30, repeat endoscopy at an interval according to the Spigelman stage Management of ampullary adenoma according to size (>1 cm), presence of high-grade dysplasia, symptoms, patient characteristics and increased rate of progression ◦ Endoscopic ampullectomy with subsequent endoscopic surveillance ◦ Surgery if endoscopically unresectable, ampullary adenocarcinoma, Spigelman stage IV duodenal polyposis or recurrence after endoscopic resection

* Endoscopic features associated with familial adenomatous polyposis and gastric cancer include gastric mounds, white mucosal patch, carpeting, >1 cm polyps, pyloric gland adenoma, gastric adenomas, and hyperplastic polyps.

**Table 2:** Overview of the clinical guidelines of upper gastrointestinal manifestations in familial adenomatous polyposis [59,60,101,102].

Overview of the Clinical Guidelines on Surveillance and Management of Upper Gastrointestinal Manifestations in Familial Adenomatous Polyposis			
European Hereditary Tumor Group-European Society of Coloproctology	National Comprehensive Cancer Network *	American Society of Gastrointestinal Endoscopy	UK Cancer Genetics Group
Start of endoscopic surveillance -Endoscopic surveillance of the upper GI tract may start after age 18, but no later than 30 years (LE: low) Surveillance examinations -Gastric surveillance intervals depend on the number, the dimensions, and the histological characteristics of adenomas (LE: low) -Post-duodenal surgery surveillance intervals depend on the type of duodenal surgery performed (LE: low) -Duodenal and papillary surveillance could rely on cap-assisted forward-viewing endoscopy for complete visualization of the papilla. If the papilla is not adequately viewed, side-viewing endoscopy should be used (LE: moderate) -Chromoendoscopy, both digital and dye-chromoendoscopy, can be used to improve the visualization of duodenal, papillary and gastric adenomas (LE: moderate) -NBI could also improve the visualization of duodenal and papillary adenomas (LE: moderate) -Video-capsule endoscopy is not adequate for gastric, duodenal and papillary surveillance (LE: low) -EUS and DBE are not part of routine endoscopic surveillance, but they could be useful as second-level diagnostic and/or therapeutic exams (LE: low) -No statement can be provided on the use of random duodenal biopsies (LE: –) -No formal recommendations to adopt or not systematic random papillary biopsies can be made (LE: very low) -Taking random biopsies of the papilla improves the diagnosis of low-grade dysplasia (LE: high) Endoscopic treatment -Endoscopic downstaging should be personalized according to endoscopic findings. Ideally, Spigelman stage IV should be downstaged as much as possible and an attempt to downstage Spigelman stage III can be performed (LE: low) -All non-papillary duodenal lesions >10 mm should undergo endoscopic resection (LE: moderate) -Non-papillary duodenal lesions measuring 5–10 mm in size could undergo either endoscopic resection or surveillance (LE: low) -All papillary adenomas should be candidates for endoscopic resection, but especially if harboring HGD, villous histology, or if >10 mm in size (LE: low) -All gastric adenomas larger >5 mm should undergo endoscopic resection (LE: low) -All gastric, duodenal and ampullary histologically proven carcinomas with endoscopic features suggestive of invasive adenocarcinoma should undergo surgery with or without systemic therapy, rather than endoscopic resection (LE: strong) Duodenal surgery versus endoscopic management -Curative surgical resection must be offered to surgically resectable, histologically proven duodenal and ampullary adenocarcinoma. (LE: strong) -Prophylactic surgical resection could be considered for Spigelman stage IV duodenal polyposis. (LE: moderate) -Prophylactic surgical resection could be considered for Spigelman stage II–III that is not endoscopically manageable (LE: low) -Papillary adenomas >10 mm or with HGD should undergo endoscopic resection, rather than surgical resection, if feasible. (LE: low) -All duodenal, papillary and gastric lesions with histologically proven invasive carcinoma should undergo surgery (if surgically completely resectable). (LE: strong) -Spigelman stages III and IV duodenal polyposis without evidence of invasive tumor should undergo endoscopic treatment, if feasible, rather than surgical resection. However, there should be a low threshold to offer surgical resection once downstaging appears no longer manageable endoscopically (LE: low) -Papillary and duodenal adenomas should undergo endoscopic resection, rather than surgery, if feasible (LE: low) -Pancreato-duodenectomy is the procedure of choice in case of suspected duodenal cancer. For prophylactic surgery, both pancreas-sparing duodenectomy and pancreatico-duodenectomy may be considered (LE: low) Management of gastric findings -Endoscopic resection of FGPs has not been demonstrated to reduce the risk of gastric adenocarcinoma. However, in cases of large or symptomatic FGPs, endoscopic resection may be considered after expert evaluation (LE: low) -Fundic gland polyposis may progress to gastric adenocarcinoma in patients with FAP. Such risk cannot be quantified up to now (LE: very low) -Endoscopic resection may be a consideration for FGPs that are large or symptomatic, after expert evaluation (LE: low) -Suspected gastric adenomas should be removed, endoscopically if feasible (LE: low) Management of small intestinal findings including post UGI surgery -After surgery, the neo-duodenum and jejunum should receive endoscopic surveillance. (LE: low) -Small bowel surveillance is not routinely indicated, but small bowel examination is recommended before duodenal surgical intervention (LE: low) -When examination of the small bowel is indicated, video-capsule endoscopy is the method of choice. If positive, patients should undergo enteroscopy for diagnosis and therapy (LE: low) Chemoprevention Insufficient evidence to support the recommendation of any chemopreventive agent for decreasing polyp size and number in the duodenum due to the lack of an acceptable risk/benefit ratio -Chemoprevention does not delay or prevent risk-reducing surgery in the upper GI tract.	Start of endoscopic surveillance -Recommend upper endoscopy (including complete visualization of the ampulla of Vater) starting at around age 20–25 years. -Consider baseline upper endoscopy earlier, if family history of aggressive duodenal adenoma burden or cancer Gastric findings and management: • Recommend representative sampling of polyps <10 mm that appear as FGP by multiple biopsies or endoscopic resection at baseline exam • Resect polyps ≥10 mm, as well as any polyps with endoscopic markers of advanced pathology or high-risk features. If there is suspicion for malignancy in a lesion, recommend referral to an expert center for management (endoscopic submucosal dissection (ESD) vs. surgery). • Recommend considering referral to an expert center for management by endoscopists with expertise in FAP for management of mounds of gastric polyps that are limiting accuracy, and resection of polyps with high-risk/advanced pathology. If other high-risk characteristics are present, consider endoscopic management to debulk proximal polyposis. • Recommend resection of all polyps in the antrum • Patients with high-risk lesions that cannot be removed by standard endoscopic techniques (including snare removal with or without EMR) should be referred to a specialized center for consideration of ESD versus gastrectomy • Gastrectomy is indicated for multifocal high-grade dysplasia and intramucosal or invasive cancer • Roux-en-Y esophago-jejunostomy reconstruction after total gastrectomy may require balloon-assisted enteroscopy for continued duodenal polyposis and ampullary surveillance •Surveillance intervals of gastric polyps are based on histology, size, and dysplasia • If partial gastrectomy is performed for antral neoplasia, then continue surveillance of the remaining stomach as indicated • Intervals for upper endoscopy surveillance should be determined based on gastric and/or duodenal findings and whichever requires more frequent surveillance should be applied. Duodenal findings and management: -Endoscopic duodenal surveillance based on modified Spigelman score and stage -For patients with advanced duodenal polyposis consider referral to an expert center for management by endoscopists with expertise in FAP. -Biopsy ampullary lesions that are concerning for neoplasia before attempted endoscopic resection -The Panel •Recommends EUS for large ampullary lesions or large duodenal polyps with features concerning for malignancy before endoscopic or surgical resection •Suggests ERCP at the time of endoscopic papillectomy to assess for evidence of extension into either the biliary or pancreatic ducts •Recommends prophylactic PD stent placement and rectal indomethacin during endoscopic papillectomy to reduce the risk of PEP •Recommends that individuals with FAP who are considering weight loss surgery be referred to an expert center for multidisciplinary discussion of bariatric interventions, taking into account the challenge of routine duodenal and gastric surveillance after RYGB surgery Refer to Guidelines from the ASGE for recommendations about the approach to sampling/removal of duodenal polyps Chemoprevention: -No FDA-approved medications -Insufficient data regarding definitive endpoints such as prevention of duodenal/ampullary cancer or need for surgery -Refer to expert centers for consideration of enrollment in clinical trial	Start of endoscopic surveillance -Recommend upper GI surveillance at 20–25 years of age or before colectomy (low quality of evidence) Surveillance examinations -Recommend upper GI surveillance based on the interval advised for the most severely affected organ, whether stomach or duodenum (low quality of evidence) -Surveillance examinations should include random biopsy sampling as well as targeted biopsy sampling of any suspicious lesions to assess for dysplasia and accurate duodenal Spigelman stage. Baseline Spigelman score ≥7 is associated with the development of duodenal HGD (low quality of evidence) Endoscopic treatment -Recommend endoscopic resection of gastric and duodenal polyps >1 cm, given the risk of developing dysplasia (low quality of evidence) -Recommend endoscopic resection of all antral polyps (low quality of evidence) -Recommend careful examination of the ampulla and periampullary region using a duodenoscope or cap-assisted gastroscope, given the predilection for cancer in this area (low quality of evidence) -Recommend biopsy sampling of the ampulla to assess for villous histology or dysplasia for only those with an identifiable mucosal abnormality, with care taken to avoid the pancreatic orifice because of the risk for pancreatitis (low quality of evidence) Chemoprevention: -Recommend the use of chemopreventive agents within the confines of a tertiary hereditary cancer center and/or as part of clinical trials (very low quality of evidence)	Start of endoscopic surveillance -Recommend upper GI surveillance for FAP patients starting at the age of 25 years (GRADE of evidence: low; Strength of recommendation: strong) -We suggest that for those considered at risk, where predictive genetic testing is not possible, screening with upper GI endoscopy is not routinely recommended but should be started if/when a clinical diagnosis of FAP is made based on colorectal phenotype (GRADE of evidence: very low; strength of recommendation: weak) Surveillance examinations -A surveillance interval determined by the combination of Spigelman classification and a staging system for ampullary disease may be the most helpful and reliably replicated clinical means of managing duodenal surveillance Endoscopic treatment -Although there are reports of chromoendoscopy increasing duodenal adenoma detection, its utility in clinical practice is not established -There are no data published regarding outcomes of endoscopic therapy for gastric adenomas in FAP. Referral to a specialist centre for assessment and management seems prudent given the lack of evidence and absence of consensus guidelines - The role of endoscopic therapy in the duodenum and ampullary is not well established. It would be most prudent for patients being considered for endoscopic therapy for duodenal disease to be referred to their local specialist centre Chemoprevention: Insufficient evidence of the benefit of chemoprophylaxis in polyposis syndromes. (GRADE of evidence: moderate; strength of recommendation: strong)

* = All National Comprehensive Cancer Network (NCCN) recommendations are Category 2A. Based upon lower-level evidence, there is uniform NCCN consensus (≥85% support of the Panel) that the intervention is appropriate.

## Abbreviations

BE, Barrett’s Esophagus; EGD, esophagogastroduodenoscopy; FAP, familial adenomatous polyposis; GC, gastric cancer; HDWL, high-definition white light; NBI, narrow-band imaging; WMP, white mucosal patch; SS, Spigelman stage.
